# Sporadic Retroperitoneal Hemangioblastoma: Report of a Case and Review of the Literature

**DOI:** 10.1155/2017/4206489

**Published:** 2017-05-18

**Authors:** F. G. Jalikis, B. L. Hoch, R. Bakthavatsalam, M. I. Montenovo

**Affiliations:** ^1^Department of Anatomic Pathology, University of Washington, Seattle, WA 98195, USA; ^2^Department of Surgery, University of Washington, Seattle, WA 98195, USA

## Abstract

We report a case of sporadic isolated hemangioblastoma arising from the retroperitoneum and provide a review of the scarce literature regarding this very rare tumor. Furthermore, we thoroughly describe the pathologic features and the broad differential diagnosis that should always be included in the study of any retroperitoneal soft tissue mass to arrive at the final diagnosis.

## 1. Introduction

Hemangioblastoma (HB) is a rare low grade neoplasm (WHO grade I) of uncertain histogenesis, characterized by the proliferation of closely packed capillaries admixed with large neoplastic stromal cells. HB most commonly arises in the cerebellum and less frequently in the brain stem, spinal cord, and supratentorium. Sporadic HB accounts for 50% to 75% of all cases and tends to present as a solitary cystic lesion with an intramural nodule within the posterior fossa, usually leading to obstructive hydrocephalus. The remaining cases (25%) occur in the setting of Von Hippel-Lindau disease (VHL) [1], an autosomal dominant disorder caused by germline mutations of the VHL gene, characterized by the occurrence of multiple neoplasms including hemangioblastomas of the brain, spinal cord, and retina, clear cell renal cell carcinoma, pheochromocytoma, neuroendocrine tumors, and renal, hepatic, and pancreatic cysts.

Extraneural HB is very rare. The first case of HB developing in the soft tissues was published by Brodkey et al., who, in 1995, described a case of HB arising in the radial nerve [2]. Extraneural HB has also been reported in the liver [3], lung [3], pancreas [4], retroperitoneum [5], kidney [6], and nasal skin [7]. Infrequently, these tumors were associated with VHL disease.

We report a case of sporadic isolated HB arising from soft tissue of the retroperitoneum with its clinicopathologic features and review of the literature.

## 2. Clinical Summary

**Figure 1:**
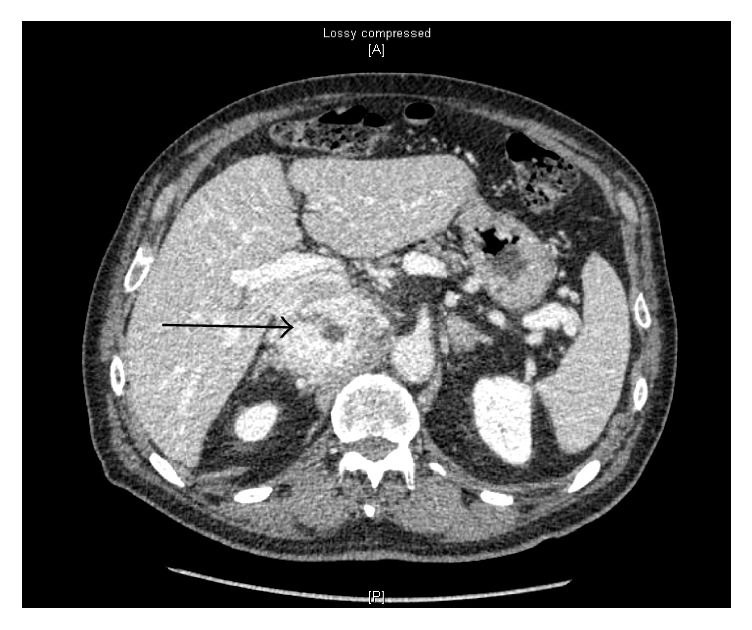
CT scan findings. 6.4 cm × 5.9 cm × 6.6 cm right retroperitoneal mass posterior to the retrohepatic inferior vena cava (arrow).

A 79-year-old gentleman with multiple cardiac comorbidities underwent a computed tomography back in 2009 for evaluation of an infrarenal abdominal aortic aneurysm. Incidentally he was found to have a retroperitoneal mass posterior to the retrohepatic inferior vena cava measuring 6.4 cm × 5.9 cm × 6.6 cm. The patient was followed up with annual imaging and in 2011 the lesion measured 7.2 cm × 5.5 cm × 6.9 cm (Figure 1) and was described as a heterogeneously enhancing mass abutting the retrohepatic inferior vena cava, with focal loss of intervening fat planes with the inferior vena cava and the left renal vein. Laterally, the mass was abutting the right adrenal gland. The kidneys were unremarkable, with no evidence of involvement. Because of the increasing size of the mass and uncertainty of the diagnosis, decision was made to proceed with surgical excision. The resection was performed through a transperitoneal open approach with a subcostal incision. At surgery, a 12.5 cm × 7 cm × 3.5 cm well circumscribed mass was identified firmly adherent to the IVC, right renal vein, celiac axis, and the right adrenal gland. The right kidney was uninvolved. The patient recovered without any complication and was discharged home.

## 3. Pathological Findings

**Figure 2:**
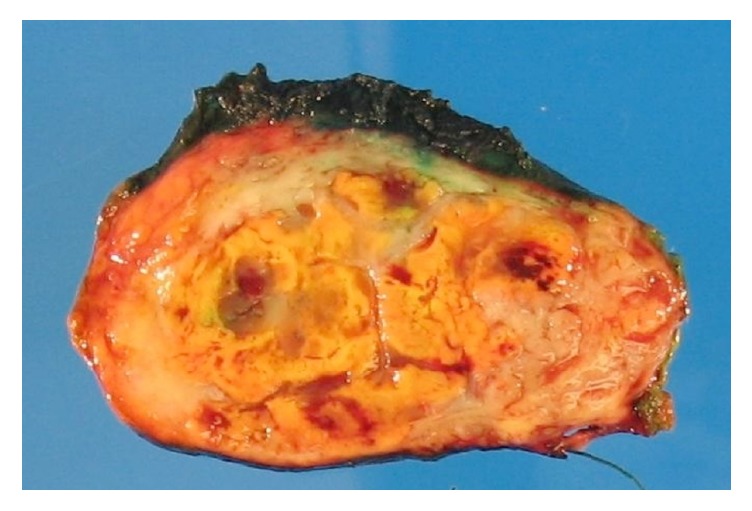
Macroscopic findings. Well circumscribed, pseudoencapsulated, 12.5 cm mass with variegated cut surfaces.

Gross examination revealed a well circumscribed, pseudoencapsulated, 12.5 cm mass with variegated cut surfaces (Figure 2). The tumor was clearly separated from the adjacent adrenal gland, which appeared grossly unremarkable.

**Figure 3:**
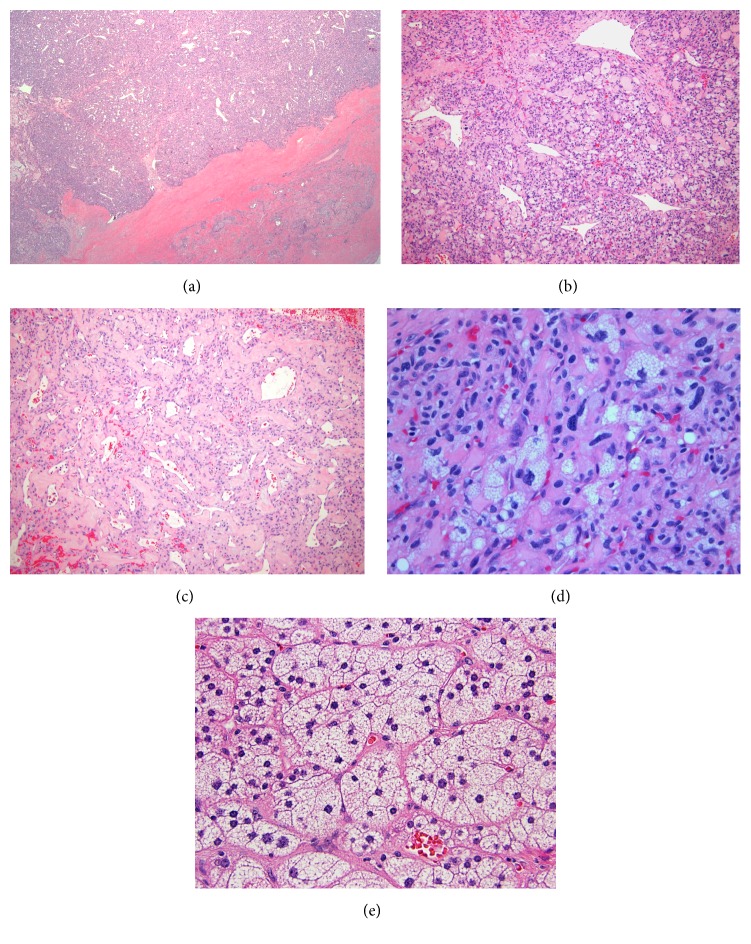
Histological examination demonstrated a well circumscribed and cellular lesion with a thin fibrous capsule ((a), H&E stain, 50x). The lesion was composed of sheets of cells with interspersed abundant capillaries and scattered large vessels ((b), H&E stain, 100x). At low power, patchy areas resembled a capillary hemangioma ((c), H&E stain, 100x). High power examination showed large neoplastic cells with foamy cytoplasm and irregular hyperchromatic nuclei, mimicking the native adrenal cortical cells, admixed with benign-appearing capillaries ((d), H&E, 400x). Native adrenal cells ((e), H&E, 200x).

**Figure 4:**
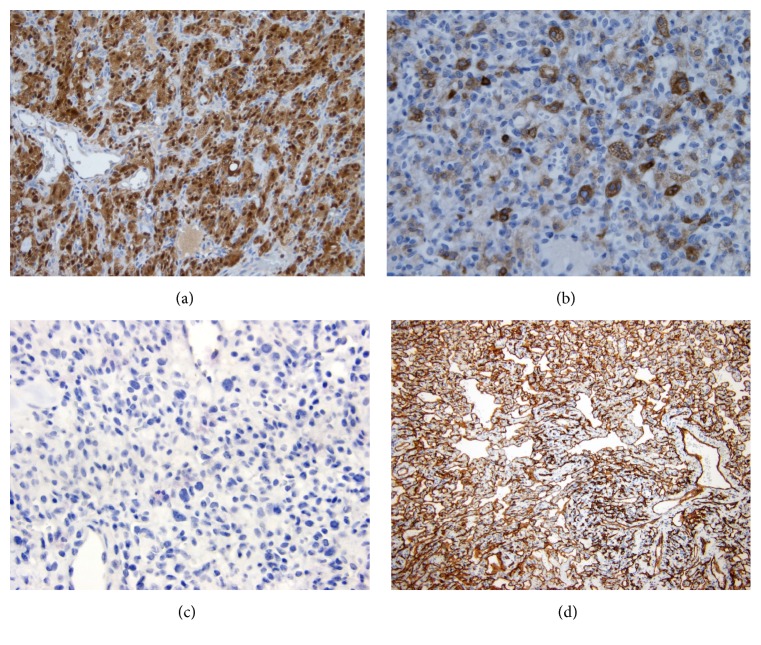
Immunohistochemical studies demonstrated diffuse S100 expression in the neoplastic cells ((a), S100 immunostain, 200x) and cytoplasmic expression of inhibin in the stromal cells ((b), inhibin immunostain, 400x). No expression of chromogranin was identified ((c), chromogranin immunostain, 400x). CD34 highlighted the prominent vascular network ((d), CD34 immunostain, 100x).

Microscopically, the neoplasm was encircled by a thin fibrous capsule (Figure 3(a)). It was composed of closely packed capillaries and large multivacuolated cells with pink or clear foamy-lipidized cytoplasm mimicking the native adrenal cortical cells (Figure 3(b)). However, in contrast to the normal adrenal cortical cells, the neoplastic nuclei appeared enlarged and focally atypical (Figure 3(c)). Patchy areas resembling capillary hemangioma were present. No mitoses were identified. Immunohistochemical studies demonstrated diffuse S100 expression in the neoplastic cells (Figure 4(a)) and cytoplasmic expression of inhibin in the stromal cells (Figure 4(b)). In addition, CD34 highlighted the prominent vascular network (Figure 4(c)). The neoplastic cells were negative for neuroendocrine, renal, melanoma, and adrenal cortical markers.

## 4. Discussion

HB is a very uncommon neoplasm, often located in the central nervous system, predominantly in the cerebellum. This tumor very rarely occurs outside the central neuraxis. After reviewing the English literature, we found a total of 12 cases of sporadic HBs arising in soft tissues, including the present case. This is the sixth case report of HB originating in the soft tissue of the retroperitoneum. All the reported cases of retroperitoneal HBs shared common features [5, 8–10]. On gross examination, they presented as well circumscribed, pseudoencapsulated masses, predominantly solid with yellow cut surfaces due to their rich lipid content. The case described by Fanburg-Smith et al. demonstrated a large cystic component, similar to that seen in the central nervous system HBs [5]. Areas of hemorrhage were also present in the majority of the cases. Histologically, these neoplasms showed features very similar to those seen in the central nervous system HBs and were characterized by a mixture of closely packed capillaries and large vacuolated stromal cells with variable nuclear atypia and rare to absent mitoses. Importantly, none of these cases appeared to involve a peripheral nerve or to arise in the setting of Von Hippel-Lindau syndrome.

Despite multiple studies, the histogenesis of the neoplastic stromal cells in HB remains obscure. Proposed origins include glial [11], endothelial [12], arachnoid [13], neuroendocrine [14], fibrohistiocytic [15], and neuroectodermal cells [16]. An additional hypothesis suggests that these neoplasms derived from embryonic cell types with divergent differentiation potential. Several findings appear to support the theory of a common ancestry, including the fact that the stromal cells express some proteins (stem cell leukemia, brachyury) characteristic of embryonic progenitor cells with hemangioblastic differentiation potential [17].

Patton et al. were the first to delineate the most important clinicopathologic features of this entity [18]. Soft tissue HB occurs more often in females (7 of the 9 non-VHL patients were women, 77%) while central HB has tendency for male predominance. Non-VHL patients with soft tissue HB tend to present 2 decades later (mean, 60 years) than VHL patients with central HB. On imaging, it usually presents as a solid mass, with only rare cases (including this one) showing a cystic component. Extraneural HB occurs most commonly in the retroperitoneum and it is not always associated with peripheral neural structures. Lastly, based upon the clinical follow-up in 7 cases, soft tissue HBs are considered benign tumors and tend to occur sporadically.

The diagnosis of soft tissue HB is very challenging, mainly because of the unexpected occurrence of this rare nervous system tumor in the soft tissue. Careful examination of the gross specimen is key to establish lack of communication with the adrenal gland and the kidney. Differential diagnosis includes metastatic clear cell renal carcinomas, paraganglioma, and pheochromocytoma. Additional considerations include lipogenic tumors with hibernomatous features, chondroid lipoma, solitary fibrous tumor, and cellular capillary hemangioma. Careful histological examination and the use of immunohistochemistry can aid in the diagnosis. Metastatic clear cell renal cell carcinoma (CCRCC) can bear a striking similarity to hemangioblastoma. CCRCC is usually positive for PAX8 and CAIX and negative for S100 and inhibin-A. Paraganglioma/pheochromocytoma expresses chromogranin-A and synaptophysin and lacks expression of inhibin in the majority of cases. S100 highlights sustentacular cells. Adrenal cortical carcinoma (ACC) also needs to be considered, especially when there is involvement of the adrenal gland. ACC is positive for MART-1, inhibin, Melan-A, and CAM 5.2 (30% of cases). STAT-6 immunostain can be useful to differentiate HB from solitary fibrous tumor (Table 1). Once the diagnosis is made, it is imperative to exclude the possibility of a neoplasm arising in the setting of Von Hippel-Lindau syndrome. In the current case, an extensive workup was negative.

In conclusion, we reported a case of extraneural HB arising from soft tissue in the retroperitoneum. Although very rare, this tumor should be considered in the differential diagnosis of retroperitoneal soft tissue tumors featuring multivacuolated cells and prominent vascular network and exhibiting cytoplasmic expression of *α*-inhibin by the stromal cells and diffuse S100 expression.

## Figures and Tables

**Table 1:** Hemangioblastoma. Differential diagnosis. (+) positive; (−) negative.

Differential diagnosis	Immunohistochemistry
Clear-cell renal-cell carcinoma	PAX8 +; CAIX +, inhibin −
Paraganglioma/pheochromocytoma	Chromogranin +, synaptophysin +, S100 + (sustentacular cells)
Adrenal cortical carcinoma	MART-1 +, calretinin +, inhibin +, Melan A +, CAM 5.2 + (30% of cases)
Solitary fibrous tumor	STAT-6+

## References

[1] Plate K. H., Vortmeyer A. O., Zagzag D. (2007). Von Hippel-Lindau disease and haemangioblastoma. *WHO Classification of Tumours of the Central Nervous System*.

[2] Brodkey J. A., Buchignani J. A., O'Brien T. F. (1995). Hemangioblastoma of the radial nerve: case report. *Neurosurgery*.

[3] Rojiani A. M., Owen D. A., Berry K., Woodhurst B., Scudamore C. H. (1991). Hepatic hemangioblastoma: an unusual presentation in a patient with Von Hippel-lindau disease. *The American Journal of Surgical Pathology*.

[4] Bird A. V., Mendelow H. (1959). Lindau's disease in a south african family a report on three further cases. *British Journal of Surgery*.

[5] Fanburg-Smith J. C., Gyure K. A., Michal M., Katz D., Thompson L. D. R. (2000). Retroperitoneal peripheral hemangioblastoma: a case report and review of the literature. *Annals of Diagnostic Pathology*.

[6] Liu Y., Qiu X.-S., Wang E.-H. (2012). Sporadic Hemangioblastoma of the Kidney: a rare renal tumor. *Diagnostic Pathology*.

[7] Boyd A. S., Zhang J. (2001). Hemangioblastoma arising in the skin. *American Journal of Dermatopathology*.

[8] Huang Y., Han X. C., Lv G. S. (2014). Sporadic hemangioblastoma of the retroperitoneum. *International Journal of Clinical and Experimental Pathology*.

[9] Yoshida A., Oda R., Shibahara J., Fukayama M., Tsuda H. (2010). Soft-tissue hemangioblastoma of the retroperitoneum: a case study and review of the literature. *Applied Immunohistochemistry and Molecular Morphology*.

[10] Nonaka D., Rodriguez J., Rosai J. (2007). Extraneural hemangioblastoma: a report of 5 cases. *The American Journal of Surgical Pathology*.

[11] Alles J. U., Bosslet K., Schachenmayr W. (1986). Hemangioblastoma of the cerebellum—an immunocytochemical study. *Clinical Neuropathology*.

[12] Jurco S., Nadji M., Harvey D. G., Parker J. C., Font R. L., Morales A. R. (1982). Hemangioblastomas: histogenesis of the stromal cell studied by immunocytochemistry. *Human Pathology*.

[13] Mizuno J., Iwata K., Takei Y. (1993). Immunohistochemical study of hemangioblastoma with special reference to its cytogenesis. *Neurologia Medico-Chirurgica*.

[14] Becker I., Paulus W., Roggendorf W. (1989). Histogenesis of stromal cells in cerebellar hemangioblastomas. An immunohistochemical study. *American Journal of Pathology*.

[15] Nemes Z. (1992). Fibrohistiocytic differentiation in capillary hemangioblastoma. *Human Pathology*.

[16] Kepes J. J., Rengachary S. S., Lee S. H. (1979). Astrocytes in hemangioblastomas of the central nervous system and their relationship to stromal cells. *Acta Neuropathologica*.

[17] Gläsker S., Li J., Xia J. B. (2006). Hemangioblastomas share protein expression with embryonal hemangioblast progenitor cell. *Cancer Research*.

[18] Patton K. T., Satcher R. L., Laskin W. B. (2005). Capillary hemangioblastoma of soft tissue: report of a case and review of the literature. *Human Pathology*.

